# Agent Based Modelling Helps in Understanding the Rules by Which Fibroblasts Support Keratinocyte Colony Formation

**DOI:** 10.1371/journal.pone.0002129

**Published:** 2008-05-07

**Authors:** Tao Sun, Phil McMinn, Mike Holcombe, Rod Smallwood, Sheila MacNeil

**Affiliations:** 1 Department of Engineering Materials, University of Sheffield, Sheffield, United Kingdom; 2 Department of Computer Science, University of Sheffield, Sheffield, United Kingdom; Tel Aviv University, Israel

## Abstract

**Background:**

Autologous keratincoytes are routinely expanded using irradiated mouse fibroblasts and bovine serum for clinical use. With growing concerns about the safety of these xenobiotic materials, it is desirable to culture keratinocytes in media without animal derived products. An improved understanding of epithelial/mesenchymal interactions could assist in this.

**Methodology/Principal Findings:**

A keratincyte/fibroblast o-culture model was developed by extending an agent-based keratinocyte colony formation model to include the response of keratinocytes to both fibroblasts and serum. The model was validated by comparison of the *in virtuo* and *in vitro* multicellular behaviour of keratinocytes and fibroblasts in single and co-culture in Greens medium. To test the robustness of the model, several properties of the fibroblasts were changed to investigate their influence on the multicellular morphogenesis of keratinocyes and fibroblasts. The model was then used to generate hypotheses to explore the interactions of both proliferative and growth arrested fibroblasts with keratinocytes. The key predictions arising from the model which were confirmed by *in vitro* experiments were that 1) the ratio of fibroblasts to keratinocytes would critically influence keratinocyte colony expansion, 2) this ratio needed to be optimum at the beginning of the co-culture, 3) proliferative fibroblasts would be more effective than irradiated cells in expanding keratinocytes and 4) in the presence of an adequate number of fibroblasts, keratinocyte expansion would be independent of serum.

**Conclusions:**

A closely associated computational and biological approach is a powerful tool for understanding complex biological systems such as the interactions between keratinocytes and fibroblasts. The key outcome of this study is the finding that the early addition of a critical ratio of proliferative fibroblasts can give rapid keratinocyte expansion without the use of irradiated mouse fibroblasts and bovine serum.

## Introduction

While the volume of data in biology is increasing rapidly with outputs from genomic, proteomic and metabolomic approaches it doesn't follow that it is easy to interpret this growing body of new data-indeed it is becoming increasingly difficult for biologists to integrate such a complexity of information without a holistic view of organisms[1]–[2]. This study follows on from a previous one from our group [3] in which we used computational modelling as a tool to improve our understanding of one particular biological system, skin cells organise into an epithelium. In this model we closely coupled *in vitro* and *in virtuo* approaches to explore different hypotheses about how normal human keratinocytes (NHK) and a transformed keratinocyte cell line (HaCat) formed colonies and achieved new insights into how keratinocytes form colonies [3].

In the current study we now move to the next level of complexity and seek to extend the previous agent based model to investigate the interactions between normal human keratinocytes and fibroblasts both *in virtuo* and *in vitro*. There is a plethora of data showing that mesenchymal cells profoundly influence epithelial cell organisation. In the culture of human keratinocytes this information has been used routinely since the initial publications by Rheinwald and Green in 1975 [4] using an irradiated feeder layer of mouse fibroblasts (i3T3) to support the attachment and proliferation of adult human keratinocytes in the presence of a mitogens rich media containing 10% bovine foetal calf serum (FCS). Despite the clinical need to avoid the use of xenobiotic agents (foetal calf serum, cholera toxin) and cells (murine fibroblasts) the use of an irradiated mouse fibroblast feeder layer in a mitogen rich media with 10% bovine serum as first used clinically in the early 1980's [5] remains the most commonly used methodology for rapid expansion of adult keratinocytes for clinical use to this day [6]. The reason for this is simple. It is a robust and reliable methodology for culturing adult human skin cells often from small initial biopsies and so far it outperforms efforts to obtain a completely defined culture approach for human keratinocyte expansion. However with growing concerns about the transmission of bovine spongiform encephalitis (BSE) from the use of bovine serum it would be desirable to culture cells under completely defined culture conditions.

Our previous research demonstrated that NHK could be expanded by co-culturing these cells with human dermal fibroblasts (HDFs) in Green's media without foetal calf serum [7]–[8]. We have also shown that human fibroblasts can perform as well as murine fibroblasts in supporting the expansion of keratinocytes and indeed keratinocytes expanded on fibroblasts in the absence of serum tended to show less differentiation than those expanded with serum [9] which is another desirable property when expanding cells for clinical use. In the original Rheinwald and Green methodology the murine fibroblasts were lethally irradiated so that they could not expand in culture (or if accidentally transferred to the patient) [4]. However, empirical data from our laboratory shows that one can get expansion of keratinocytes on non-irradiated fibroblasts to satisfactory levels if one pays attention to the ratio between the fibroblasts and keratinocytes [7].

Agent-based modeling is a computational approach that simulates the interactions of autonomous entities (agents, or individual cells) with each other and their local environment to predict higher level emergent patterns. Outputs of these models can be visual and easily accessible to biologists (which facilitates interdisciplinary collaboration) and models can be built more quickly and at lower cost than laboratory experiments, freeing resources for a more informed exploration of the hypothesis space [3]. Our aim in this study was to take our recently established agent based model of keratinocyte colony expansion and extend it to look at the interactions between keratinocytes and fibroblasts to test hypotheses of how fibroblasts interact with keratinocytes to promote keratinocyte colony formation.

Our approach was to use the extensive literature on keratinocyte/fibroblast interactions combined with *in vitro* experimentation (comparing murine and human fibroblasts) to generate an initial rule set for defining fibroblast behaviour. This was then incorporated into the previous model for keratinocytes in monoculture and the model was adapted as necessary to simulate the macroscopic morphogenesis of NHKs and fibroblasts *in vitro*. The model was validated by comparison of the *in virtuo* model with *in vitro* multi-cellular behaviour of NHKs and HDFs both in single and co-culture conditions in Green's medium in the presence and absence of serum. The robustness of the model to simulate the multicellular morphology of NHKs and various types of HDFs in co-culture was also tested by varying various properties of HDF (such as proliferation rate, differentiation rate and motility). The model was then used to propose a range of hypotheses to explain the *in vitro* behaviour of these two cell types. Analysis of the model demonstrated that the proliferation and differentiation of NHK would be influenced by the initial ratio of HDF to NHK, the proliferation rate of the HDF and the timing of when HDF were introduced to NHKs. From these hypotheses we then focused on those which could be examined with an *in virtuo/in vitro* comparison. Thus, specifically, we looked at to what extent the ratio of fibroblasts to keratinocytes would promote colony formation and whether introducing fibroblasts prior to keratinocyte differentiation would accelerate keratinocyte colony formation to a greater extent than if these were introduced when keratinocytes had begun to differentiate. We also examined whether proliferative fibroblasts would be more effective than growth arrested fibroblasts in supporting keratinocyte colony formation and throughout we examined to what extent the presence of fibroblasts would allow one to dispense with the inclusion of foetal calf serum.

This research demonstrated that a closely integrated *in virtuo* and *in vitro* approach is a powerful tool for understanding complex biological systems. Both *in virtuo* simulation and *in vitro* experimentation indicated that: 1) the ratio of fibroblasts to keratinocytes would critically influence the rate of keratinocyte colony expansion, 2) this ratio needed to be optimum at the beginning of the co-culture, 3) proliferative fibroblasts would be more effective than irradiated cells in expanding keratinocyte colonies and 4) in the presence of an adequate number of fibroblasts, keratinocyte colony expansion would be independent of serum.

### Development of the Agent Based Model

In the following sections, the agent based modelling approach is briefly introduced. We then summarize the biological literature which was abstracted to derive the rule sets for the agents for the co-culture model. Finally, our in virtuo predictions and in vitro experiments of the interactions between keratinocytes and fibroblasts are described.

### Concept of agent-based modelling

The concept of the NHK-HDF co-culture model is similar to our previous agent-based NHK colony formation model [3]. Briefly, the model is composed of two parts: the *agents* (representing *cells*), and the *environment* (representing the culture dish in which the cells reside, along with global factors such as growth factors). Each cell was modelled as a non-deformable sphere (20 µm in diameter) governed by a rule set, and cells were capable of migration, proliferation and differentiation. In this study, the culture dish was modelled as a user-defined flat square surface (500 µm×500 µm) with a wall (100 µm high) around it.

The following is the agent rule sequence as summarized in Table 1. Initially, agents (*cells*) output their location and type (NHKs, which are then subdivided into stem cells, transit amplifying (TA) cells, committed cells or corneocytes or HDFs, which are subdivided into proliferative or differentiated HDFs) to the message lists for other cells to read. Each cell then performs rules specific to its own position in the cell cycle. Following this, cells follow instructions to change to another cell type based on the differentiation rules. Cells then execute their migration and physical rules. All rules are executed in the context of the agent's own internal state and its immediate environment as discovered through interrogation of the message lists. The time step of each iteration in the co-culture model is 30 minutes. The model framework used is that of Coakley [10]. (FLAME, http://www.flame.ac.uk). The framework and a detailed user manual of the framework are freely available for users to download.

**Table 1:** Agent rule sequence performed by each cell in each iteration.

1. Output location and cell type to the message lists
2. Cell cycle & proliferation rules
3. Differentiation rules
4. Migration rules
5. Physical rules

### Interactions between HDF and NHK

In a normal healthy human epidermis, the achievement and maintenance of the equilibrium between proliferation and cell loss of NHK is precisely controlled by various mechanisms [11]–[12]. Our previous NHK colony formation model [3] mainly focused on the autoregulation of NHK colony formation [13]–[17]. However, the complex dermal-epidermal interaction is another crucial regulation mechanism. This is based on interactions between fibroblasts and keratinocytes which encompass soluble factors, extracellular matrix (ECM), and direct cell-cell contacts [11], [18]–[24]. The literature describing these which is used for this study and the biological rules abstracted from this literature are reviewed as follows.

### HDF-NHK interactions through soluble factors

A variety of soluble factors produced by dermal/epidermal cells exert complex regulatory effects on both producer and recipient cells [19], [25]–[26]. This is evident as a different range of cytokines and growth factors are found to be expressed by NHKs at different stages of culture or in different proliferation/differentiation states [24]. NHKs not only stimulate fibroblast proliferation by producing platelet-derived growth factor (PDGF), basic FGF (FGF-2) [13], IL-1α and 1β [19], but also inhibit the growth and induce senescence and apoptosis of fibroblasts through active synthesis and turnover of cell-permeable ceramide [27]. Reciprocally, fibroblasts can influence the proliferation/differentiation state of NHK through soluble factors [28]. This complex interaction can be illustrated by a well established double paracrine mechanism: NHKs release IL-1 [11], [21], [29]–[30] and or parathyroid hormone-related protein (PTHrP) [21], [31] to induce the expression of growth factors such as KGF, GM-CSF and IL-6 in HDF, which in turn regulate both NHK proliferation and differentiation [21], [30]. The expression ratio of KGF and GM-CSF in fibroblasts is controlled by two AP-1 transcription factors (c-Jun and Jun B) [20]–[21], [32]. IL-1 can also induce the expression of PGE2 in fibroblasts, which will suppress the expression of IL-1 in NHK [33]. This novel type of mutually induced signalling circuits probably has functional significance *in vivo* [19], [30].

The complex influence of soluble factors on NHK can also be demonstrated by the spectrum of cytokines that have varied but overlapping functions on the proliferation/differentiation of NHK [12]–[13], [34]. For example, EGF and TGF-α both stimulate NHK proliferation; KGF mediates NHK proliferation compatible with differentiation [16], [35]–[38] **;** GM-CSF induces NHK differentiation compatible with proliferation, while TGF-β has various functions on NHK [39]–[40]. Although KGF is the most important ligand to bind to the KGF receptor, other KGF-R ligands such as FGF10 [21], [41] and FGF22 [37], [42] have been found to compensate for the lack of KGF [16], [19], [43]. Moreover, different factors function through different mechanisms. For example, both TGF-α and TGF-β affect NHK through paracrine and autocrine pathways, while KGF is exclusively a paracrine modulator [36], [38].

In this research, all of these soluble factors were simply divided into stimulatory and inhibitory factors and simulated implicitly. For example in the early stage of the cell cultures the literature shows that both NHKs and HDFs tend to express more stimulatory factors, while more inhibitory factors are produced in late stage cultures. Serum, which contains several stimulatory factors, was modelled explicitly as a single stimulatory global factor.

### HDF-NHK interactions through ECM

The fibroblast is the main cell type involved in neodermis formation which it does through depositing ECM components in a dialogue with NHK regulated through a delicate balance between synthesis and degradation [25], [44]–[45]. There is a dynamic interaction and co-operation between epithelial and mesenchymal cells in all aspects of ECM deposition and subsequent structural organization. At various stages of wound healing ECM undergoes transient changes to induce remarkable changes in cell phenotype [11], [18], [46]–[48]. For example, the expression of collagen IV and laminin 1 are reciprocally stimulated in both NHK and fibroblasts [48]–[49].

In this co-culture model, ECMs produced by NHKs were implicitly simulated using an autoregulation mechanism as in our previous model [3]. However, the NHK induced ECM expression of HDF was modelled explicitly: Thus direct cell-cell contact between NHK and HDF was modelled as inducing the expression of ECM in HDF to coat the tissue culture surface (changing the colour of the model surface), NHKs then responded to the ECM produced by the HDF by being more proliferative when they attached to the coated surface.

### HDF-NHK interactions through direct cell-cell contact

In human skin NHKs and HDFs are separated by the basement membrane (BM), thus rendering epidermal-mesenchymal cell-cell contact mediated mechanisms less possible [19]–[20]. However, in *in vitro* co-culture direct contact of both cells is desirable [50], as it can significantly enhance the expression of cytokines [51] such as epimorphin in fibroblasts [52] and IL-6 in both cells [53]. Since cell-cell contact can not only inhibit [54]–[56] but also enhance cell proliferation [57]–[58], it should be considered in a broader biological context [59]–[60]. Before confluence, cultured fibroblasts move apart and most of the cells are proliferative; after confluence, fibroblasts stop dividing due to contact or density dependent inhibition of cell division [61]–[63]. The proliferation of NHK can be stimulated or inhibited by direct NHK-HDF contact in co-culture [44].

In our co-culture model the mutual stimulatory/inhibitory influences between NHKs and HDFs in direct contact were modelled explicitly. Proliferative HDFs (P-HDFs) stimulated the attachment and proliferation of proliferative NHKs (i.e. stem cells and transit amplifying cells) by direct cell-cell contact. Similarly direct contact with proliferative NHKs enhanced the proliferation of P-HDFs, while direct contact with differentiated NHKs induced rapid differentiation of all HDFs.

### The heterogeneity of fibroblasts

Fibroblasts are the most commonly cultured but also possibly the most poorly understood cells, for the term ‘fibroblast’ covers a very heterogeneous cell population [64], differing in their morphology and their ability to proliferate, synthesize macromolecules and contract collagen gel [22], [32], [65]. HDFs are in a ‘resting’ or inactive state in normal skin but in an injured tissue they change their phenotype quickly, migrate to the wound area, proliferate and produce ECMs to repair the injury [18], [24], [61], then differentiate into myofibroblasts which play crucial roles in wound contraction, tissue remodeling and permanent healing [18], [45], [66]–[67]. Because of this heterogeneity, the characterization of fibroblast populations before their use *in vitro* has been recognized as a real challenge [68]. In our *in virtuo* model, all dermal fibroblasts were described as active proliferating cells or inactive non-proliferative differentiated fibroblasts (D-HDF) for simulation purposes.

### Irradiated 3T3 mouse fibroblasts and human dermal fibroblasts

NHK proliferation can be stimulated or inhibited by fibroblasts in co-culture [44]. To prevent any inhibitory effects of the overgrowing of proliferative fibroblasts and thus inhibiting the expansion of NHK colonies, also to avoid the transfer of proliferative mouse fibroblasts to the patient's wound (if accidentally transferred to the patient), several methods of growth arresting fibroblasts have been used. Specifically gamma irradiation, mitomycin C treatment and H_2_O_2_ treatment have all been employed to prepare growth arrested post-mitotic fibroblasts as feeder cells [9], [19], [44], [53], [69]. Growth arrested fibroblasts were reported to lose contact inhibition but to contribute a combination of mitogens and matrix proteins to enhance the expansion of NHK colonies [44], [47], [53]. During this process the NHK colonies push aside and finally eliminate these growth arrested fibroblasts. This phenomenon might also be responsible for the diminished IL-6 production reported in some older co-cultures in addition to the possible down regulation of IL-1 receptor expression on these feeder cells [53].

In our *in vitro* experiments time lapse microscopy indicated that less than 50% of the seeded i3T3s or iHDFs attached to the tissue culture surface. These attached cells did not proliferate or migrate and most of them died out gradually during the first week of culture in Green's medium minus FCS (G-FCS). In contrast, more than 95% of non-growth arrested HDFs attached readily and achieved confluence within 5–7 days but then gradually died out within 8–14 days in G-FCS media. (In Green's medium plus FCS (G+FCS), non-growth arrested HDFs survived for months at high density). Therefore, the irradiated fibroblasts were simulated *in virtuo* by knocking out the dividing and migrating rules for the HDFs. Their ability to attach to the substrate was also weakened based on our *in vitro* observations and on the literature [19], [53]. Both irradiated mouse fibroblasts and irradiated HDF were simulated using the same set of biological rules without introducing any rules to distinguish between them.

### Application of HDF and NHK co-culture rules

The biological rules related to NHK cell cycle, proliferation, differentiation and migration that were as in the previous keratinocyte colony formation model [3] and were kept unaltered in the co-culture model are summarized in Table 2. The literature used to derive these rules and the details of these rules were described previously [3] but are briefly reiterated as follows.

**Table 2:** Summary of biological rules used to govern agent behaviours in the NHK colony formation model.

	Cell cycle	Proliferation	Differentiation	Migration
**Stem cell**	60h	Divide into 2 stem cells. EX: Contact with stem cells, within stem cell colony. IM: Stimulatory factors, ECM	Differentiate to TA cells. EX: Outside stem cell colony, contact inhibition, detached from substrate, Ca++, contact with committed cells, corneocytes. IM: Inhibitory signals such as FasL, ceramide	Passive migration. EX: Forces between cell and cell, cell and substrate.
**TA cell**	30h	Divide into 2 TA cells. EX: Contact with stem cells, TA cells, within TA cell colony. IM: Stimulatory factors, ECM.	Differentiate to committed cells. EX: Outside TA cell colony, contact inhibition, detached from substrate, Ca++, contact with committed cells, corneocytes, remaining in G0 for certain period of time. IM: Inhibitory signals such as FasL, ceramide	Passive migration. EX: Forces between cell and cell, cell and substrate. Active migration. EX: 1 µm/min
**Committed cell**	None	Does not proliferate	Differentiate to corneocytes. EX: Contact with corneocytes, remaining in committed cell state for certain period of time. IM: Inhibitory signals such as FasL, ceramide.	Passive migration. EX: Forces between cell and cell, cell and substrate.
**Corneocyte**	None	Does not proliferate	Fully differentiated-no further differentiation possible.	Passive migration. EX: Forces between cell and cell, cell and substrate.

**Notes**: EX: explicitly modeled rules, IM: implicitly modeled rules. TA cells: transit amplifying cells, ECM: extracellular matrix.

NHK stem cells can attach to the culture surface, proliferate, form tight colonies, and control the size of the stem cell colony. When the stem cell colony reaches a certain size, the stem cells on the colony edge will differentiate to TA cells. TA cells can migrate, divide, stratify and control the size of the TA cell colony. When TA cells are a certain distance away from stem cells, they will differentiate to committed cells. Committed cells gradually lose their nuclei and further differentiate to corneocytes. Both committed cells and corneocytes can send out differentiation/death signals to TA and stem cells. Stem and TA cell will differentiate to committed cell when they receive the differentiation/death signals. To develop the co-culture model, biological rules concerning the inclusion of fibroblasts and serum were derived and integrated into the keratinocyte colony formation model, which will be explained in the following sections. All the biological rules that relate to cell cycle, proliferation, differentiation and migration of NHKs and fibroblasts in the co-culture model are also summarized in Table 3.

**Table 3:** Summary of biological rules used to govern agent behaviours in the NHK/HDF co-culture model.

	Cell cycle	Proliferation	Differentiation	Migration
**Stem cell**	60h	Divide into 2 stem cells. EX: Contact with stem cells, within stem cell colony, presence of serum and/or HDF, contact with P-HDF, coated substrate. IM: Stimulatory factors.	Differentiate to TA cells. EX: Outside stem cell colony, contact inhibition, detached from substrate, Ca++, contact with committed cells, corneocytes, absence of serum and/or HDF. IM: Inhibitory signals such as FasL, ceramide	Passive migration. EX: Forces between cell and cell, cell and substrate.
**TA cell**	30h	Divide into 2 TA cells. EX: Contact with stem cells, TA cells, within TA cell colony, presence of serum and/or HDF, contact with P-HDF, coated substrate. IM: Stimulatory factors.	Differentiate to committed cells. EX: Outside TA cell colony, contact inhibition, detached from substrate, Ca++, contact with committed cells, corneocytes, remaining in G0 for certain period of time, absence of serum and/or HDF. IM: Inhibitory signals such as FasL, ceramide	Passive migration. EX: Forces between cell and cell, cell and substrate. Active migration. EX: 1 µm/min
**Committed cell**	None	Does not proliferate	Differentiate to corneocytes. EX: Contact with corneocytes, remaining in committed cell state for certain period of time, absence of serum and/or HDF. IM: Inhibitory signals such as FasL, ceramide.	Passive migration. EX: Forces between cell and cell, cell and substrate.
**Corneocyte**	None	Does not proliferate	Fully differentiated-no further differentiation possible.	Passive migration. EX: Forces between cell and cell, cell and substrate.
**P-HDF**	20h	Divide into 2 P-HDFs. EX: Contact with stem cells, TA cells, P-HDF, presence of serum and or stem cells and TA cells. IM: Stimulatory factors.	Differentiate to D-HDFs. EX: Contact inhibition, detached from substrate, contact with committed cells, corneocytes, D-HDFs, remaining in G0 for certain period of time, absence of serum and/or stem cells, TA cells. IM: Inhibitory signals such as FasL, ceramide.	Passive migration. EX: Forces between cell and cell, cell and substrate. Active migration. EX: 1 µm/min
**D-HDF**	None	Does not proliferate	Apoptosis or detachment of D-HDFs. EX: Detached by NHKs, remaining in D-HDF state for certain period of time.	Passive migration. EX: Forces between cell and cell, cell and substrate. Active migration. EX: 1 µm/min

**Notes**: EX: explicitly modeled rules, IM: implicitly modeled rules. TA cells: transit amplifying cells, P-HDF: proliferative human dermal fibroblast. D-HDF: differentiated human dermal fibroblast, ECM: extracellular matrix.

### Proliferation rules

In the co-culture model, the presence of serum has a strong stimulatory influence on proliferative NHKs (i.e. stem and TA cells) and proliferative fibroblasts. Both proliferative NHK and P-HDF stimulate each other to become more proliferative. P-HDFs proliferate and re-enter the cell cycle after every cell division until they are contact inhibited or receive differentiation signals. Thus a P-HDF agent interrogates the message lists to find the number of neighbours in its immediate vicinity. If the immediate neighbour cells are more than a certain number (*x*), then the cell enters G0. When the number of neighbouring cells falls below *x* again, the cell leaves G0 and begins a countdown to division. If however the cell stays in G0 longer than a certain period of time it changes to a differentiated HDF and these D-HDFs are modelled as having no stimulatory or inhibitory influence on NHKs. The intrinsic cell cycle designated to HDF is 20 hours (40 iterations) based on literature values [70]–[72].

### Differentiation rules

The following differentiation mechanisms were modelled for fibroblasts: (1) contact inhibition [2]; (2) presence of differentiation/apoptosis signals, such as ceramide [27] and Fas/L-Fas [73], (3) loss of cell-matrix contact [74]–[78]; (4) remaining in a quiescent state (G0) for longer than a certain period of time [79]. Most of the NHK/HDF differentiation rules still centre on NHK colony formation. In addition to an auto-regulation mechanism, the NHK colony size and the differentiation process are also subject to regulation by serum and HDF. Our *in vitro* time lapse experiments indicated that few NHKs can attach, proliferate or form NHK colonies on a normal tissue culture plastic surface when single cultured in serum free Greens media (G-FCS). However, the presence of HDFs can help NHKs to survive in G-FCS and even form bigger colonies. The influence of serum and HDF on NHK was implemented in the co-culture model as follows: Apart from the auto-regulated keratinocyte colony formation mechanism, NHK stem cells also check for the presence of serum and HDF. With serum present, NHKs can attach to the substrate and form colonies as modelled previously. The presence of HDF, especially the presence of immediate P-HDF neighbours, can help NHKs to attach and form colonies in G-FCS. The more immediate P-HDF neighbours available or the more contacts they make with P-HDF, the more proliferative the stem cell will be. Consequently the stem cell colony will be bigger. If the number of stem cell contacts is relatively low (indicating it is on the edge of the culture), the stem cell will differentiate into a TA cell.

TA cells undertake a similar process as to whether they should differentiate into a committed cell by monitoring the presence of serum and P-HDF. Both serum and P-HDF have stimulatory effects on TA cells. Stem and TA cells also slightly increase the proliferative capacity of P-HDF. Both committed cells and corneocytes send out differentiation signals (e.g. ceramide or Fas-L) to the neighbouring cells, P-HDFs differentiate to D-HDFs when they receive these signals. D-HDFs have no stimulatory effects on either stem or TA cells. (See Table 3 for a summary of these rule sets).

### Strength of cell binding and migration rules

Bond strengths between HDF and substrate are modelled as relatively weak compared with NHK-substrate bonds since HDFs can be easily pushed or detached by NHK as demonstrated by time-lapse video microscopy. As HDF were uniformly distributed on the tissue culture surface and no tight HDF colonies were observed in *in vitro* cell culture especially when the cultures were subconfluent, repelling forces were thus applied among HDFs. Repelling forces were also applied between HDF and NHK, since time lapse experiments also demonstrated contact induced inhibition of movement between HDFs and NHKs.

As the actions of cell migration and division are modelled as discrete steps that are applied to each agent individually there is a possibility that the simulated cells may overlap on the virtual culture plate. In this case, a corrective repulsive force is applied in order to push the cells apart. This is proportional to the overlapping area (a higher force for a bigger overlap). If there is no free surface remaining for cells to move to then an apoptosis mechanism is applied to HDFs so that essentially the modelled HDFs are lost from the model.

The essential mechanisms responsible for cell migration are mainly a cell density driven pressure passive movement [80]–[81] as well as a cell active movement. The cell passive movement is caused by cell-cell or cell-substrate interactions and mitotic activity. Accordingly a cell passive movement was applied to each agent corresponding to the forces acting on it. An active migration speed of approximately 1 µm/minute was also applied to HDF based on time lapse experiments and literature [70]. (Summarised in Table 3).

### Apoptosis rules

Single cultures of HDF in G-FCS showed that HDF viability decreased rapidly after culture for 5–7 days [7]–[8]. Therefore, the absence of serum in single HDF culture was assumed to cause HDF apoptosis. Time lapse experiments of HDF-NHK co-cultures demonstrated that NHK colonies were surrounded by HDF and that the NHK colonies push the HDF away. No single HDFs were observed inside NHK colonies. Accordingly, the apoptosis or detachment of HDF was explicitly modelled as follows-after a direct contact with NHK, the HDF will be pushed away by NHK. If there is no free surface for HDF to migrate to, the HDF agent will then be removed from the model by simulated apoptosis or detachment.

The code for this model will be made available from: http://www.flame.ac.uk.

## Results

### Validation of the NHK-HDF co-culture model

The model was initially established to simulate the *in vitro* multicellular behaviours of NHKs and HDFs co-cultured in Green's media in the presence and absence of FCS. Some of the biological rules related to the distribution of both types of the cells and the apoptosis of fibroblasts were verified experimentally before being incorporated into the model. To distinguish keratinocytes from fibroblasts for *in vitro* studies a combination of immunostains were used–all cell nuclei were stained with DAPI, phalloidin-TRITC staining for F-actin was used to help distinguish between the different morphologies of keratinocytes and fibroblasts, and pancytokeratin was used to specifically identify keratinocytes.

**Figure 1:**
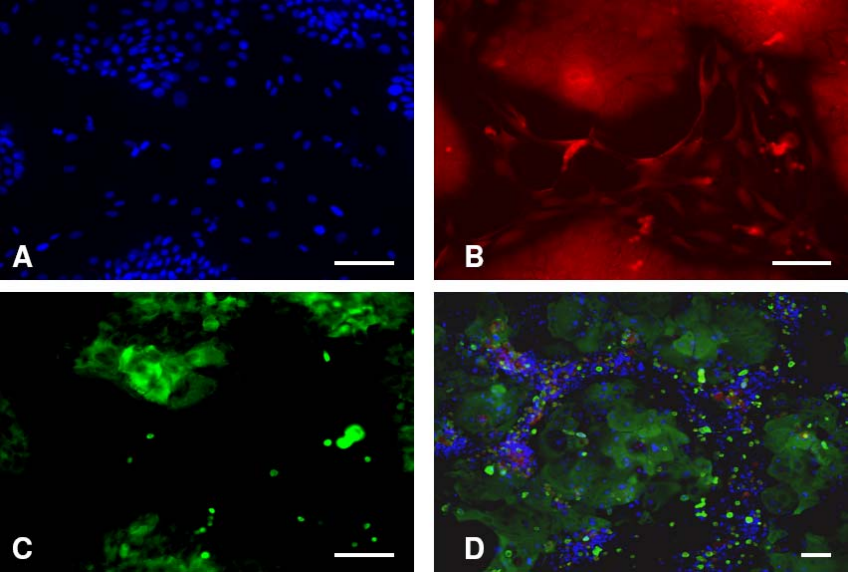
Morphologies and distributions of keratinocytes and fibroblasts in *in vitro* cultures. Normal human keratinocytes (NHKs) and human dermal fibroblasts (HDFs) co-cultured in Greens media minus fetal calf serum (G-FCS) for 5 (A-C) and 7 (D) days. In (A) all cell nuclei were stained with DAPI (blue), in (B) F-actin of cells was stained with phalloidin-TRITC (red) and in (C) NHKs were stained with a pan-cytokeratin antibody (green). In (D) keratinocytes and fibroblasts were co-cultured in G-FCS for 7 days and then exposed to live dead staining. Live cells were identified with Hoechst 33342 (blue), dead cells stained with propidium iodide (red) and cultures were then stained with a pan-cytokeratin antibody (green) to identify NHK. Bar = 100 µm.

Figure 1 shows co-cultures of NHK and HDF in serum free Green's media. Figures 1A–C show the appearance of cells co-cultured for 5 days and Figure 1D the appearance of cells co-cultured for 7 days. Examining co-cultures at 5 days one can see that there were small compact keratinocyte colonies surrounded by HDFs. By 7 days (Figure 1D) keratinocyte colonies were larger, fibroblasts were located at relatively high density surrounding these colonies and one can see that the dead cells (Red) present were mainly in the fibroblast population close to keratinocyte colonies, which confirmed the biological rules related to the distribution and apoptosis of fibroblasts.

**Figure 2 pone-0002129-g002:**
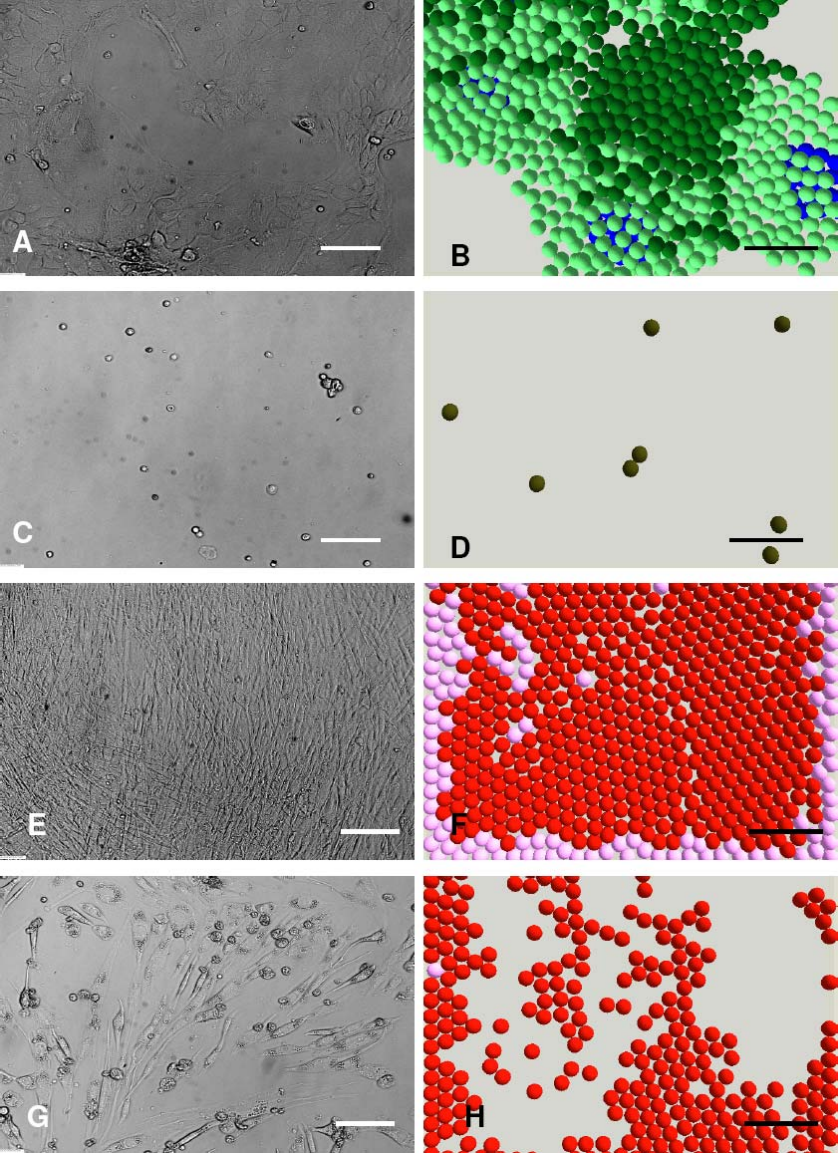
*In vitro* monocultures and the corresponding simulations of keratinocytes and fibroblasts. *In virtuo* simulations of the growth of normal human keratinocytes (NHKs) and human dermal fibroblasts (HDFs) in monocultures in the presence and absence of foetal calf serum (FCS). (A) and (C) show micrographs of NHKs single cultured for 8 days in Greens medium with and without FCS respectively, while (B) and (D) show the corresponding model simulations. (E) and (G) show micrographs of HDFs single cultured for 8 days in Greens media with or lacking FCS, while (F) and (H) show the corresponding *in virtuo* simulation. The initial numbers of keratinocyte stem cells and fibroblasts were both defined as 10 within the user-defined flat surface (500 µm×500 µm). In the agent based model different colours were used to represent keratinocyte stem cells (blue), TA cells (light green), committed cells (dark green), corneocytes (brown), proliferative fibroblasts (pink) and differentiated fibroblasts (red). Bar = 100 µm.

To validate the agent based NHK-HDF co-culture model, the simulated multicellular morphologies of HDFs and NHKs in single and co-cultures in Green.s media with and without FCS were compared with the corresponding *in vitro* morphologies. As shown in Figure 2, the colony formation of NHKs in Green's medium plus FCS (G+FCS) (Figure 2A) was reproduced in the *in virtuo* simulation (Figure 2B). For NHK in monoculture lacking FCS (G-FCS) hardly any cells survived by 8 days (Figure 1 C). NHK stem cells in G-FCS were modelled to rapidly withdraw from the cell cycle and differentiate to committed cells and then become corneocytes due to the lack of FCS (Figure 2D).

Time lapse experiments indicated that HDFs could initially survive and proliferate to confluence after culture for 5–7 days in Green's media both with and without FCS. As the cultures continued to 8–14 days, HDFs in G+FCS remained confluent (Figure 2E), while the cells in G-FCS gradually died out (Figure 2G). The influence of serum on the proliferation/apoptosis of HDFs was simulated by the agent based model as shown in Figure 2F and H. Fibroblasts were depicted as proliferative (pink) or differentiated (red) in this simulation.

**Figure 3:**
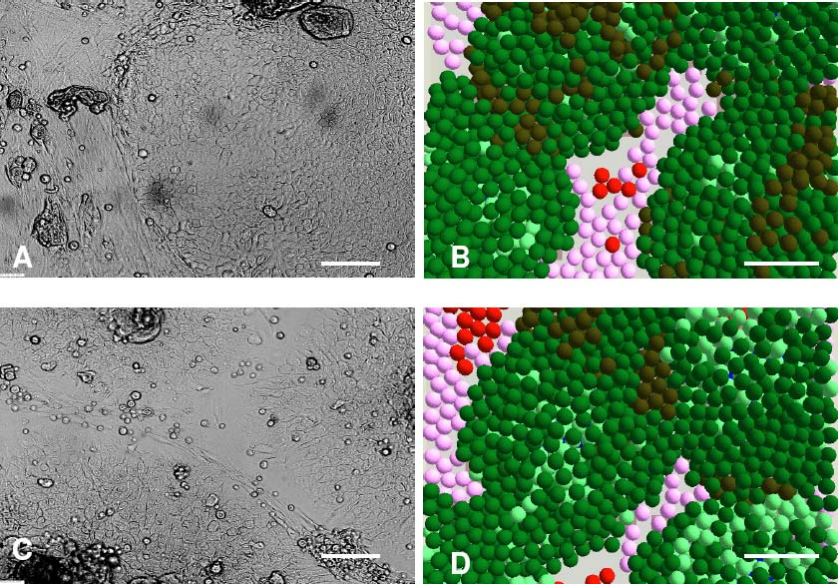
*In vitro* co-cultures and the corresponding simulations of keratinocytes and fibroblasts. (A) and (C) show micrographs of normal human keratinocytes (NHKs) and human dermal fibroblasts (HDFs) co-cultured for 8 days in Greens medium with and without foetal calf serum (FCS) respectively, while (B) and (D) show the corresponding model simulations. For modelling purposes the initial numbers of keratinocyte stem cells and fibroblasts were both defined as 10 within the user-defined flat square surface (500 µm×500 µm). In the agent based model different colours were used to represent keratinocyte stem cells (blue), TA cells (light green), committed cells (dark green), corneocytes (brown), proliferative fibroblasts (pink) and differentiated fibroblasts (red). Bar = 100 µm.

In co-culture, the absence of FCS had relatively little effect on the expansion of keratinocyte colonies due to the dynamic interactions between NHKs and HDFs. The macroscopic morphogenesis of NHKs and HDFs co-cultured in Greens media with or without FCS were compared using both *in vitro* and *in virtuo* models over 8 days as shown in Figure 3 and very similar results were obtained in terms of multicellular morphologies and the percentage of the cell culture surface which was occupied by keratinocyte colonies.

In summary, Figures 1–3 show that computational models of NHK and HDF in single or co-cultures in Green's media in the presence and absence of serum could be made to reflect the *in vitro* multicellular behaviour of both NHKs and HDFs.

### Testing of the in virtuo model robustness for investigation of NHK-HDF interactions

Fibroblasts are a very heterogeneous cell population, differing in their morphology and ability to migrate, proliferate and synthesize ECM [22], [32], [65], [64]. Because of this it was necessary to test whether the co-culture model was robust enough to accommodate heterogeneous fibroblast populations. Therefore, initial *in virtuo* investigations were carried out to test to what extent the biological parameters of the fibroblasts could be changed without affecting their influence on NHKs.

**Figure 4:**
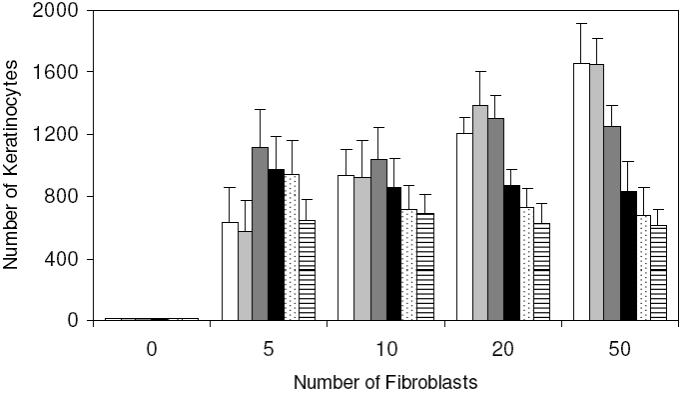
Model analysis of the effects of fibroblast density and proliferation rate on keratinocyte expansion. Within the user-defined flat square surface (500 µm×500 µm), the initial keratinocyte stem cell number in all the simulations was defined as 10, while the initial fibroblast number varied from 0 to 50. The fibroblast proliferation rates simulated were: 0% (white column), 10% (light grey column), 30% (dark grey column), 50% (black column), 70% (dotted column) and 90% (lined column). Simulation results shown are mean±SD (n = 10).

The parameters we changed were cell cycle length (from 0 to 30 hours) proliferation status (from non-proliferative to 90% proliferative), differentiation rate (from non-differentiative to 100% differentiative) and migration rate (from 0 to 1um/min). All of these fibroblast conditions resulted in a very similar macroscopic morphology and distribution of NHKs and fibroblasts-that is, NHKs and fibroblasts had distinctive multicellular morphologies with NHKs forming tight colonies, while fibroblasts were distributed throughout the areas that were not occupied by NHK colonies (as represented in Figure 3B), suggesting that the model is robust enough to simulate different populations of fibroblasts. It should be emphasized however that changing these fibroblast parameters did have significant effects on the proliferation and differentiation of NHK in particular affecting the time taken to achieve a useful NHK expansion. For example, different proliferation rates of fibroblasts can cause different NHK productivities as shown in Figure 4. Accordingly the model is very useful to investigate the influences of different parameters of fibroblasts biology on keratinocyte colony expansion.

Our previous *in vitro* research showed that as expected fibroblasts and serum both influenced NHK culture in Greens medium [7]–[8] but serum free culture could be readily achieved if sufficient fibroblasts were present. The desire to avoid the use of bovine serum for culture of keratinocytes for clinical use was the motivation behind these earlier studies. Accordingly the *in virtuo* model was now used to systematically investigate the spatial-temporal interactions between NHKs and HDFs both in the presence and absence of FCS. Simulations using fibroblasts with various cell cycles, proliferation/differentiation rates and motilities indicated that the maximum stimulatory influence of HDFs on NHK proliferation occurred mainly on the edges of NHK colonies and most importantly needed to occur before proliferative NHKs (i.e. stem cells and TA cells) differentiated to committed cells. The model analysis predicted that various fibroblast parameters (such as increasing HDF migration rate, changing the tendency of HDF to migrate towards NHKs and decreasing the HDF-substrate bond strength) can significantly enhance NHK colony expansion. The challenge however was to test some of these predictions and because of this our *in virtuo* research efforts were next focused on approaches that could be tested experimentally.

### Influence of fibroblast density and proliferative status on keratinocyte colony formation

Varying numbers of HDFs (0, 5, 10, 20 and 50) were co-seeded with 10 keratinocyte stem cells in a defined model area (500 µm×500 µm) and the co-cultures in G-FCS were simulated using the computational model. For each simulation with a defined initial HDF-NHK ratio, the division probability of fibroblasts was also varied within a broad range (0–90%). The simulation results indicated that although the macroscopic morphogenesis and distribution of NHKs and fibroblasts were similar, the changing fibroblast proliferation rate had a significant influence on the production of keratinocytes. As shown in Figure 4, in the absence of HDFs barely any NHKs survived to form visible colonies. In the presence of fibroblasts both the initial HDF seeding density and the HDF proliferation status influenced the proliferation (and subsequent differentiation) of NHKs. When the proliferation rate of HDFs was low (0–10%), there was a linear relationship between the initial HDF density and NHK expansion as can be seen from Figure 4. Essentially a relatively high ratio of fibroblasts to keratinocytes was needed to get good keratinocyte expansion. However as the HDF proliferation rate was increased to 30%, the initial fibroblast density did not significantly influence the outcome for keratinocyte expansion as shown in this model. As the fibroblast proliferation rate was increased further to 50, 70 and 90% and yet more fibroblasts were added (see 50 initial fibroblasts in Figure 4) then it became clear that a high ratio of fibroblasts at a high rate of proliferation actually yielded less keratinocytes overall. Thus the model analysis indicated that the fibroblast heterogeneity (exhibited as cells undergoing different rates of proliferation) should also be considered when deciding on the initial HDF seeding density to support higher rates of NHK proliferation. Our time–lapse experiments clearly indicated that less than 10% of fibroblasts were undergoing division at any time, therefore a proliferation rate of 3% was used for the proliferative fibroblasts in all the following simulations.

### Simulation of the influence of post-mitotic fibroblasts on keratinocyte expansion

**Figure 5:**
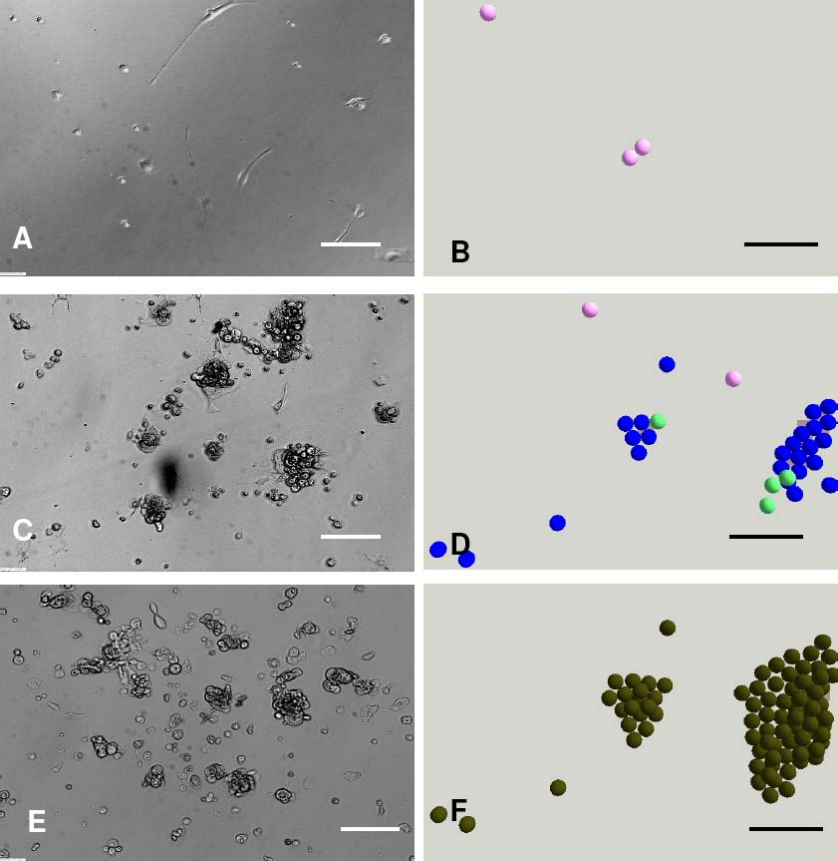
*In vitro* cultures and the corresponding simulations of keratinocytes and irradiated 3T3 fibroblasts. (A) Irradiated 3T3 fibroblasts (i3T3s) cultured for 8 days in Greens minus fetal calf serum (G-FCS) and (B) the corresponding model simulation. (C) Normal human keratinocytes (NHKs) and i3T3s co-cultured in G-FCS for 2 days and (E) 8 days respectively, (D) and (F) are the micrographs of the corresponding model simulations. The initial numbers of keratinocyte stem cell and i3T3 were both defined as 10 for either single cultures or co-cultures within the user-defined flat square surface (500 µm×500 µm). In the agent based model different colours were used to represent keratinocyte stem cells (blue), TA cells (light green), committed cells (dark Green), corneucytes (brown) and irradiated fibroblasts (pink). Bar = 100 µm.

To further investigate the influence of fibroblast proliferation status on keratinocyte expansion, post-mitotic (non-dividing) fibroblasts were simulated using the computational model. Time-lapse video microscopy of irradiated 3T3 mouse fibroblasts (i3T3s) and irradiated HDFs (iHDFs) (both are post-mitotic fibroblasts) single cultured in G-FCS showed that less than 50% of the seeded cells managed to attach and then spread on tissue culture plastic in the absence of serum (as illustrated in Figure 5A for i3T3s). As the culture progressed, no obvious cell proliferation and migration was observed and the attached cells gradually died out over 7–8 days. In contrast, more than 95% of the seeded HDFs cells attached and proliferated to confluence within 5–7 days and then gradually died out somewhere between 8–14 days in G-FCS media as mentioned earlier.

Based on our research and published literature [19], [53], the following modifications of HDF rules were made to model these irradiated fibroblasts: the proliferation and migration mechanisms were knocked out, the substrate adhesion capability was deliberately reduced and a self-detachment mechanism was introduced so that approximately 70% of the attached cells would detach from the model surface within one week. The above modifications were then verified by comparing multicellular morphologies of i3T3s obtained using both computational and biological models as shown in Figure 5A–B. The modified co-culture model was then used to simulate co-culture of NHKs and irradiated fibroblasts in G-FCS and it was found that it was necessary to introduce more i3T3s compared to HDFs due to the inability of the former to attach and proliferate.

Model analysis demonstrated that NHK stem cells initially attached and formed small tight colonies with the help of co-seeded i3T3s in G-FCS (Figure 5D). As the cultures progressed, however, these NHK colonies gradually lost their momentum to expand due to the loss of feeder cells (Figure 5F), which was confirmed experimentally (Figure 5C and E). Similar results were obtained for irradiated HDFs both in single culture and in co-culture with NHKs (results not shown).

### Supplementation of irradiated fibroblasts to enhance NHK production in G-FCS

**Figure 6:**
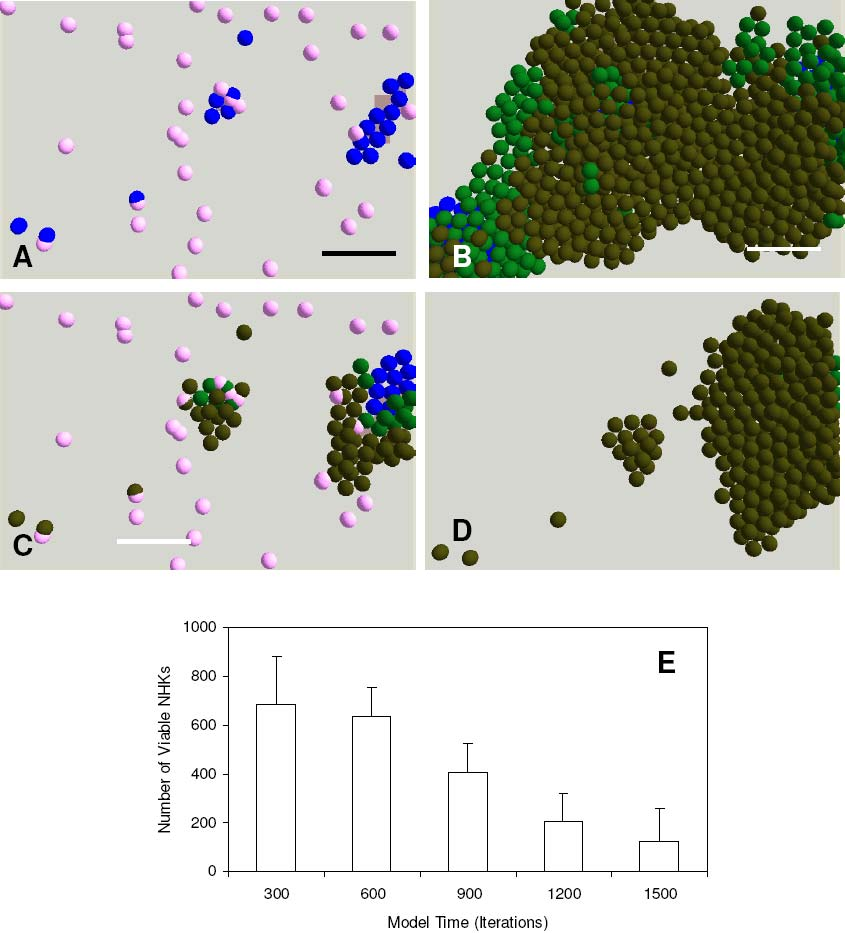
Model analysis of the supplement of extra irradiated fibroblasts when cocultured with keratinocytes. Extra irradiated fibroblasts (50) were added to a co-culture of keratinocytes and irradiated fibroblasts in serum free Greens media (A) before the keratinocyte stem cells started to differentiate and then (B) simulated for 7 more days. In (C) the extra irradiated fibroblasts (50) were supplemented after the keratinocytes had started to differentiate and then (D) simulated for a further 7 days. In the agent based model different colours were used to represent keratinocyte stem cells (blue), TA cells (light green), committed cells (dark green), corneocytes (brown) and irradiated fibroblasts (pink). Bar = 100 µm. (E) shows the numerical effect of adding extra irradiated fibroblasts at different time points (iterations in the model) on keratinocyte proliferation. The simulation results shown are mean±SD (n = 10). The initial numbers of keratinocyte stem cells and irradiated fibroblasts in all the above simulations were both defined as 10 within the user-defined flat square surface (500 µm×500 µm), while 50 extra irradiated fibroblasts were supplemented at the times (iterations) indicated.

To optimize the co-culture of NHKs with irradiated fibroblasts for higher NHK productivity in serum free media, the modelling approach was used to explore different hypotheses. A feeder layer supplement strategy was explored in which the gradual loss of irradiated fibroblasts during culture could be compensated for by supplementing NHK with extra feeder fibroblasts during the culture. Further model analysis showed that the extra feeder cells needed to be added before the proliferative NHKs (stem cells and TA cells) differentiated to committed cells, otherwise the stimulatory effects of the supplemented cells on NHK production would be very limited as illustrated in Figure 6A–E.

**Figure 7:**
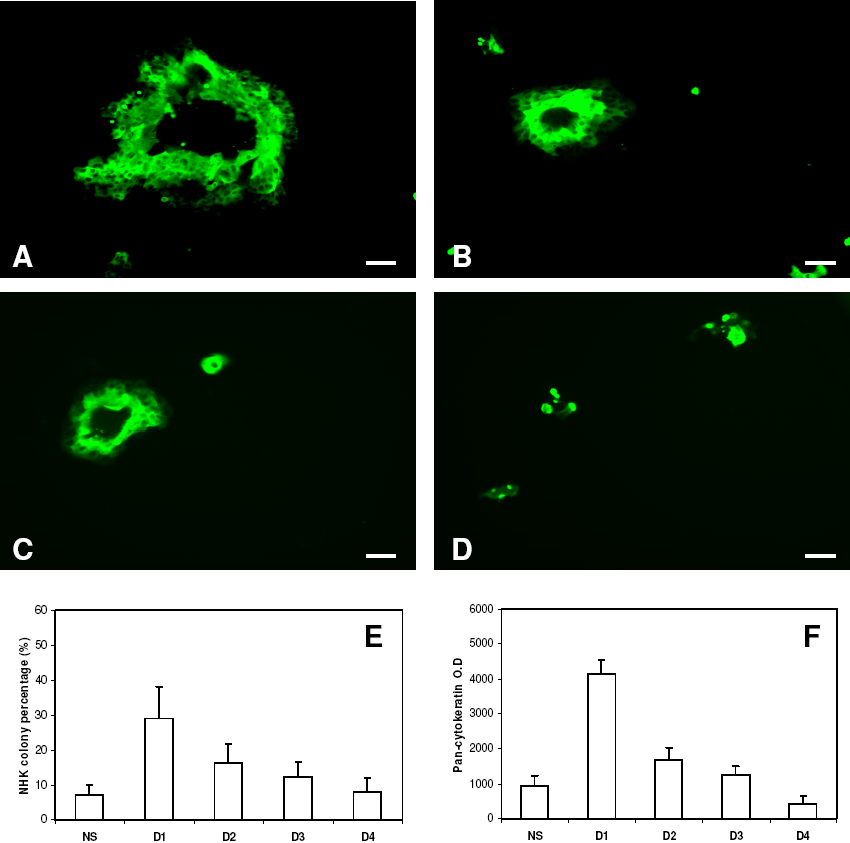
*In vitro* analysis of the supplement of extra irradiated mouse fibroblasts when cocultured with keratinocytes. (A-D) Micrographs of normal human keratinocytes (NHK) co-cultured with irradiated mouse fibroblasts (i3T3) in serum free Greens medium when extra i3T3s were not supplemented (NS) during the culture, supplemented at day 1(A), 2 (B), 3 (C) or 4 (D) respectively and cultured for 12 days. Bar = 100 µm. Effect of the addition of extra i3T3s at different culture time periods on (E) the percentage of the tissue culture well occupied by NHK colonies after culture for 7 days and (F) the expression of pan-cytokeratin in NHKs (assessed as an indirect indicator of keratinocyte mass)after culture for 12 days. Results shown are mean±SD (n = 3).

To confirm this prediction, *in vitro* experiments were then designed and performed using NHKs and i3T3s. In order to avoid the influence of any HDFs present in freshly isolated NHKs, passaged NHKs (1×10^5^ cells per well) and i3T3s (5×10^4^ cells per well) were co-seeded in each well of 24 well plates in G-FCS. Cultures were then divided into five groups, one of which received no further fibroblasts (referred to as NS -not supplemented) or were supplemented with extra i3T3s (1×10^4^ cells per well) at days 1, 2, 3 and 4 respectively. The NHK colonies in all cultures at day 7 were measured for their percentage occupancy of the culture surface using a non-invasive image analysis methodology. After culture for 12 days, cultures were terminated and fixed for pan-cytokeratin immunofluorescent staining to give an indirect assessment of total keratinocyte mass. As demonstrated in Figure 7A–F, the earlier the supplement of i3T3s was given the higher the percentage of the culture surface was occupied by NHK and the total NHK mass obtained. Supplementation with extra i3T3s at day 1 resulted in the highest NHK productivity while the same cell number supplemented at day 4 led to no significant increase in NHK production compared to non-supplemented cells. These results strongly supported the model prediction.

**Figure 8:**
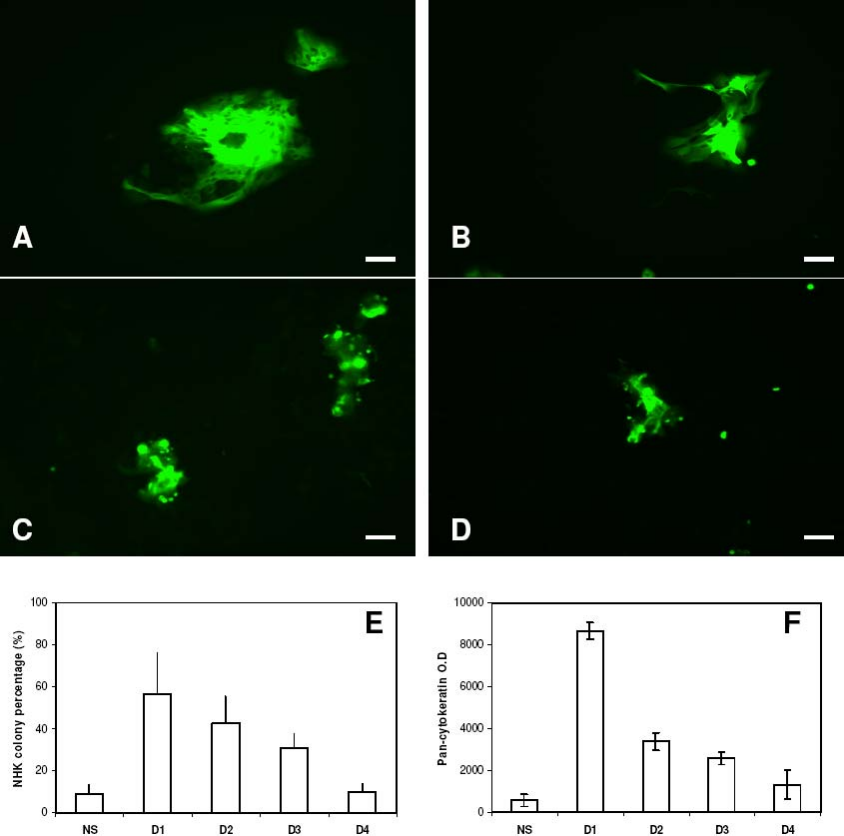
*In vitro* analysis of the supplement of irradiated human dermal fibroblasts when cocultured with keratinocytes. (A-D) Micrographs of normal human keratinocytes (NHK) co-cultured with irradiated human dermal fibroblasts (iHDF) in serum free Greens medium when extra iHDFs were not supplemented (NS) during the culture, supplemented at day 1(A) ,2 (B), 3 (C) and 4 (D) respectively and cultured for 12 days. Bar = 100 µm. Effect of the addition of extra iHDFs at different culture time periods on (E) the percentage of the tissue culture well occupied by NHK colonies after culture for 7 days and (F) the expression of pan-cytokeratin in NHKs ( assessed as an indirect indicator of keratinocyte mass )after culture for 12 days. Results shown are mean±SD (n = 3).

Since no extra biological rules were used to distinguish i3T3 from iHDF in the co-culture model, the supplement strategy predicted by the model should be also valid for the co-culture of iHDF and NHK and similar *in vitro* supplement experiments were conducted using iHDFs. As illustrated in Figure 8A–F, iHDFs supplemented at early culture significantly increased NHK productivity compared to the same cells added to late cultures.

### Exploration of the differences between growth arrested and non-growth arrested fibroblasts for their ability to support keratinocyte colony formation

**Figure 9:**
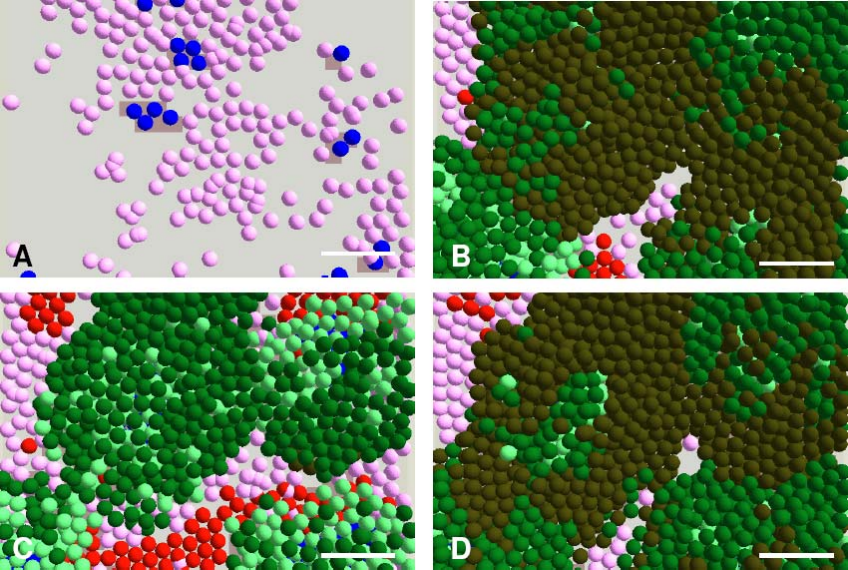
Model analysis of the supplement of human dermal fibroblasts when cocultured with keratinocytes. Extra human dermal fibroblasts (HDFs) were added to a co-culture of keratinocytes and HDFs in serum free Greens media (A) before the keratinocyte stem cells started to differentiate and then (B) simulated for 7 more days. In (C) the extra HDFs were added after the keratinocytes had started to differentiate and then (D) simulated for a further 7 days. The initial numbers of keratinocyte stem cell and fibroblast were both defined as 10 within the user-defined flat square surface (500 µm×500 µm), while 50 extra HDFs were supplemented for each simulation. In the agent based model different colours were used to represent keratinocyte stem cells (blue), TA cells (light green), committed cells (dark green), corneucytes (brown), proliferative fibroblasts (pink) and differentiated fibroblasts (red). Bar = 100 µm.

**Figure 10:**
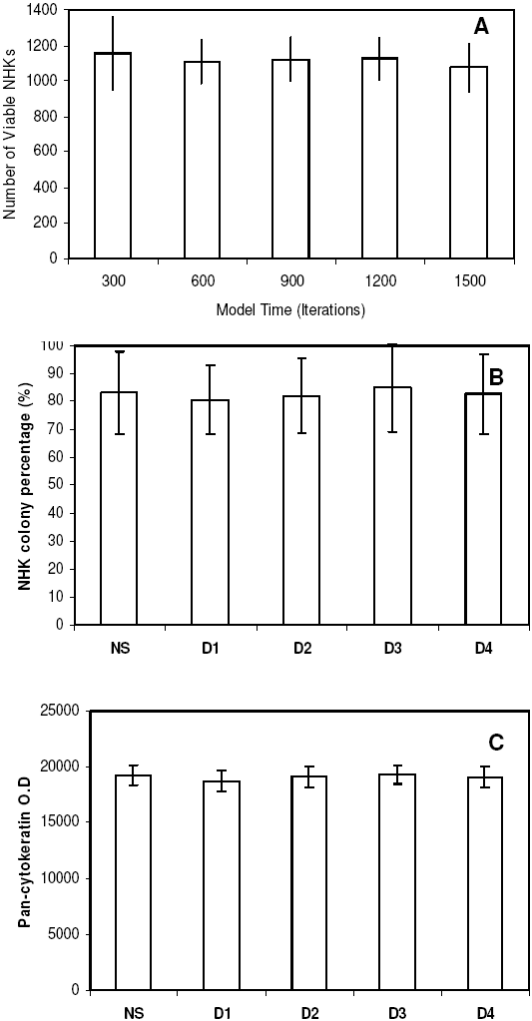
*In vitro* and *in virtuo* analysis of the supplement of dermal fibroblasts when cocultured with keratinocytes. (A) Effect of the addition of human dermal fibroblasts (HDFs) at different time points (iterations in the model) on keratinocyte proliferation. The initial numbers of keratinocyte stem cell and fibroblast were both defined as 10 within the user-defined flat square surface (500 µm×500 µm), while 50 extra fibroblasts were added for each simulation at different simulation time (iterations). Simulation results shown are mean±SD (n = 10). Effect of the addition of extra HDFs at different culture time periods on (B) the percentage of the tissue culture well occupied by NHK colonies after culture for 7 days and (C) the expression of pan-cytokeratin in NHKs (assessed as an indirect indicator of keratinocyte mass )after culture for 12 days. Results shown are mean±SD (n = 3).

Clearly irradiated fibroblasts differed significantly from non-irradiated fibroblasts both in their ability to proliferate and also in the extent to which the irradiated fibroblasts progressively detached from the surface under serum free culture conditions. Accordingly the supplementation strategy for adding HDF to NHKs was next analyzed using the computational model before it was applied to real cell cultures to see to what extent HDFs might differ to irradiated HDFs in supporting keratinocyte colony expansion. Systematic simulation indicated that additional HDFs whether supplemented before or after the proliferative NHKs differentiated had no further effects on NHK productivity, providing enough HDFs were initially available to help the initial stem cells to attach and proliferate on the substrate as shown in Figure 9A–D and Figure 10A. Bearing both in mind the final modelling exercise addressed the influence of supplementing cultures with HDF and here simulations predicted that as shown in Figure 9 whether HDF were supplemented early or late made no significant difference to the NHK colony expansion. Further *in virtuo* analysis demonstrated that this appeared to be because the HDF density outside the NHK colonies increased steadily as the co-culture progressed since HDFs were able to proliferate and migrate in contrast to the irradiated fibroblasts. It was also apparent in these cultures that as the NHK colonies expanded rapidly with the help of HDFs there was a decreasing area available for the HDFs, which also increased the local HDF density.

To check the validity of these predictions, passaged NHKs (1×10^5^ cells per well) and HDFs (1×10^4^ cells per well) were co-seeded in 24 well plates using G-FCS. As before cultures were also divided into different groups, one of which was not given any further HDFs and others were given 1×10^4^ extra HDFs per well on days 1, 2, 3 and 4 respectively. After culture for 7 days the percentage of keratinocyte colonies occupying the culture surface was assessed and then at 12 days all cultures were terminated and pan-cytokeratin was used to get an indication of total keratinocyte mass. The results are shown in Figure 10 and here it can be seen that adding extra HDF at days 1 through to 4 had no further effect on the keratinocyte colonies on the culture surface (Figure 10B) or overall keratinocyte mass (Figure 10C) as predicted by the *in virtuo* modelling. However, the key point to note is that keratinocyte expansion on HDF was far superior to NHK expansion on iHDF or i3T3's even when these cells were initially added at 5 times higher density. Thus as summarised in Table 4 at least 80% of the culture well was occupied with keratinocyte colonies (Figure 10B) within 7 days if these were initially co-cultured with proliferative HDFs at a ratio of 1 HDF to 10 NHK. A later supplement (after 24 hours) of further HDF did not imrove on this already high rate of NHK expansion, In contrast if i3T3 and iHDF cells were used at a ratio of 5 cells to 10 NHK then the rate of NHK expansion was poor (<10%) but could be improved to 30–55% by the addition of extra fibroblasts at 24 h (as shown in Figure 7E)). In summary, the data provide a logical case for using proliferative fibroblasts rather than irradiated fibroblasts to drive keratinocyte colony formation.

**Table 4:** Comparison of the ability of proliferative versus growth arrested fibroblasts to support keratinocyte colony growth in the absence of fetal calf serum.

Fibroblast Type	Initial fibroblast Density	Initial NHK Density	Area of well occupied by NHKs	Addition of more fibroblasts after 24h	Area of well occupied by NHKs
**i3T3**	5×10^4^	1×10^5^	<10%	1×10^4^	30%
**iHDF**	5×10^4^	1×10^5^	<10%	1×10^4^	55%
**HDF**	1×10^4^	1×10^5^	80%	1×10^4^	80%

## Discussion

The regulation of epidermal homeostasis involves a complex interplay between different generic and genetic mechanisms, making it difficult for biologists to investigate except by focusing on separate discrete aspects. Fortunately, computational modelling approaches can handle this complexity [1]–[2], [82]–[83] and agent-based modelling in particular is gathering popularity with biologists [84]. The idea of this modelling approach is to abstract away micro-level details to find a minimal set of properties sufficient to simulate the local interactions of components of a system to produce a global behaviour model which then allows the testing of hypotheses and the designing of new informative experiments.

Previously we developed an extensible agent-based NHK colony formation model [3]. In the current study, biological rules of serum, HDF and their influence on NHK were abstracted from an extensive published literature and our own *in vitro* experiments and then incorporated into the model to simulate the complex interactions between epidermal-dermal cells. The validity of the co-culture model was tested by comparing the simulation results of HDFs and NHKs in single or co-cultures in different environments with corresponding *in vitro* experimentation. The results from both *in virtuo* and *in vitro* models indicated that the crucial roles of serum and fibroblasts in the production of NHK were successfully simulated by the co-culture model.

As the literature indicates that fibroblasts actually represent a very heterogeneous cell population [22], [32], [64]–[65], several biological parameters (cell cycle length, the rates of proliferation, differentiation and migration) were varied *in virtuo* to examine whether the model was robust enough to simulate the different behaviour of fibroblasts. Our criterion for judging the robustness of the model was whether the multicellular morphology of NHK-HDF co-culture could still be simulated when these fibroblast parameters were varied within broad ranges. Our results demonstrated that changing the parameters within very broad ranges or knocking-out some of rules of fibroblasts all ultimately resulted in very similar macroscopic morphology and distribution of NHKs and fibroblasts but the time to achieve NHK expansion was affected as was keratinocyte expansion. In this study the co-culture model was used to explore different hypotheses of NHK-HDF interactions to optimize the speed of NHK expansion for higher productivity for clinical use. Many more *in virtuo* studies could now be undertaken with this model to examine for example how important the different variables are to the expansion of NHK.

From a modelling perspective the exercise of designing an agent based approach to understanding fibroblast/keratinocyte interactions allowed us to formulate and test hypotheses *in virtuo* some of which we could then test *in vitro*. The main “deliverable” of this study is a rational explanation for the differing behaviours of proliferative versus growth-arrested fibroblasts in supporting keratinocyte expansion. Also as it would be good to avoid the use of bovine serum when culturing cells for clinical use, a key focus in our integrated *in virtuo* and *in vitro* approaches was the examination of NHK/HDF co-culture under serum free conditions.

Simulation of the temporal-spatial NHK-HDF dynamic interactions indicated that the presence of HDF on the edges of the auto-regulated NHK colonies at different culture periods was crucial for maximum NHK production. Model analysis indicated that the initial HDF/NHK ratio had a significant influence on the proliferation/differentiation of NHK, which confirmed our previous *in vitro* research [7]. The model also predicted that the rate of fibroblast proliferation would have a major influence on NHK colony production. This has not been directly examined before to the best of our knowledge.

Ever since the seminal work of Rheinwald and Green in 1975 describing a method to culture adult human keratinocytes using irradiated murine feeder fibroblasts and a mitogen-rich media [4], feeder cells (i3T3s and iHDFs) have usually been used as post-mitotic fibroblasts [9], [19], [53], [69] to culture NHKs for both research and clinical purposes. To model these lethally irradiated cells three changes were made to the model of normal fibroblasts- proliferation and migration mechanisms were knocked out and a new rule of self-detachment based on literature findings was introduced [19]. The model was validated by comparing the behaviour of i3T3 and iHDF cells *in virtuo* with their behaviour in *in vitro* time lapse experiments in single culture in serum free Greens medium. The modified co-culture model was then used to simulate the co-culture of NHK and irradiated fibroblasts in G-FCS and the limitations of these feeder cells to enhance NHK production were explored *in virtuo*. Basically most of the irradiated cells in the presence of expanding NHK colonies were unable to survive for long (1 week) due to the detachment applied by the expanding NHK colonies and the self-detachment of the irradiated cells [19], [53].

The model predicted that the addition of more feeder cells would assist the continuing expansion of NHK colonies but that this would only work for keratinocytes in a proliferative phase. *In vitro* experiments were then designed to examine to what extent the prediction would hold true. As predicted adding extra i3T3s and iHDFs at an early keratinocyte culture stage resulted in significantly higher NHK productivity than if these cultures were supplemented with fibroblasts once keratinocytes had begun to differentiate.

The *in virtuo* effort was then refocused on the optimization of HDF-NHK co-culture and the same supplementation strategy was tested. Simulation results indicated that as NHK colonies expanded during co-culture, the HDF density outside NHK colonies increased steadily due to HDF division and the diminished area for HDF. Thus the model predicted that adding additional HDFs would not achieve any further benefit in terms of increased colony expansion, which was also confirmed by subsequent *in vitro* supplement experiments.

In summary, in this study we have described the development of an agent based NHK-HDF co-culture model. The model was developed through close coupling of *in virtuo* and *in vitro* experimentation. It was then used to generate and test different hypotheses about the rate of NHK colony production. Most importantly, it is ready to be used by practical experimentalists in a predictive sense to design and guide new informative *in vitro* experiments. Clearly, this co-culture model is still a simplification of the complex process of NHK homeostasis, thus it is based primarily on the concept of NHK autoregulation in 2D cultures in the laboratory (as we previously described [3]) and the ability of fibroblasts to provide factors (mitogens and attachment proteins) which facilitate and help accelerate this process to the extent that there is no need to provide FCS. The model does not go into detail on which attachment factors or mitogens are produced during the fibroblast/keratinocyte co-culture, neither does it attempt to address the issues of 3D cell spatial interactions to describe how these cells would interact in normal skin organisation. (Skin cells also interact with melanocytes-and respond to wounding and trauma such as UV radiation etc-again all challenges for the future).

Although this model of cell/cell interactions is still at an early stage, the current study demonstrates the synergy between computational and experimental models and shows the potential of such models to become a powerful tool for understanding complex biological systems at a system level and guiding *in vitro* research. Specifically we hope this agent based model finds value as a new investigative tool for studying epidermal biology. Access to this software model can be obtained via our website as mentioned earlier.

## Materials and Methods

### Cell Culture

The mouse fibroblasts used in this research are an established cell line-J2 3T3 cells originally obtained from Professor Howard Green, USA. Normal human keratinocytes and fibroblasts were isolated from split thickness skin (STS) harvested from theatre specimens removed following routine plastic surgical abdominoplasties and breast reductions skin under a licence (Licensing Number 12179) granted under Section 16(2)e(ii) of the Human Tissue Act 2004 (‘the Act’) by the Human Tissue Authority (UK).. Fully informed written consent was obtained from each patient prior to operation with explicit permission that removed tissue could be used for research purposes. The methodologies of cell isolation, media preparation and cell culture were as described previously [7]–[8].

### Fibroblast growth arrest

Both mouse and human dermal fibroblasts were growth arrested by lethally irradiating cells as described previously [9], [69]. Briefly, lethal irradiation was carried out on 90% confluent cultures of fibroblasts passaged into 20 ml culture media. Mouse fibroblasts (3T3 cells) were subjected to a gamma irradiation dose of 60 Gy, while human dermal fibroblasts were subjected to 200 Gy (IBL 437C Gamma Irradiator, CIS Biointernational, Burgess Hill, United Kingdom). The irradiated cells were then suspended in FCS with 10% DMSO (Sigma) and chilled at 1°C per minute to −80°C before storage in liquid nitrogen. Before use cells were thawed at 37°C and then resuspended in prewarmed growth media and plated at an appropriate density on tissue culture plate.

### Immunofluorescence microscopy for multicellular morphology

Various immunostaining methods were combined to distinguish NHK and HDF: cell nuclei were labelled with DAPI (300 nM, Vector Laboratories Inc., Ca, USA) to locate all the cells. F-actin was stained with phalloidin-TRITC (25 ug/ml, P195, Sigma-Aldrich) to help distinguish between different cell morphologies. Pan-cytokeratin immunostaining was used to specifically identify NHKs.

In brief, after removal of the culture medium, the cells in single or co-culture in tissue culture plates were washed gently with PBS (×3), fixed in 4% (w/v) paraformaldehyde for 30 minutes and then labelled with DAPI and phalloidin-TRITC for 1 h as described recently [85]. After washed thoroughly (×3 PBS), NHKs in the cultures were then specifically identified using pan-cytokeratin immunostaining [7]–[8]. After a final wash (×3 PBS), all fluorescently stains were visualized using an ImageXpressTM system (AXON, USA) and fluorescence micrographs of the labeled cells were taken using epifluorescent illumination at *λ*ex = 358 nm, *λ*em = 461 nm (for DAPI/nuclei visualization), *λ*ex = 550 nm, *λ*em = 650 nm (for phalloidin-TRITC/cell morphology visualization) and λex = 495 nm, λem = 515 nm (for FITC/Pancytokeratin visualization).

To quantify the pan-cytokeratin expression level in NHKs, all the fluorescent stain was eluted by incubating the cells in 0.1 M NaOH for 1 hour at 37°C, transferred to a cuvette and measured using a fluorescence plate reader (λex = 495 nm, λem = 515 nm).

### Assessment of cell viability

In order to confirm the detachment of HDFs on the edges of NHK colonies, a cell viability assay and pan-cytokeratin immunostaining were combined to identify live/dead cells and NHKs respectively. Briefly, after co-culture for 7 days, cells were stained with 8 µM Hoechst 33342 (Sigma Chemical Company Ltd) and 5 µM propidium iodide (Molecular Probes, Cambridge Biochemical, Cambridge UK) for 15 min at 37°C. The culture medium was then removed and the cells were washed gently with PBS (×3), fixed in 4% (w/v) paraformaldehyde for 30 minutes. NHKs in the cultures were then specifically identified using pan-cytokeratin immunostaining [7]–[8]. After a final wash (×3 PBS), all the fluorescently stained cells were visualized using an ImageXpressTM system (AXON, USA) and fluorescence micrographs of live/dead cells and NHKs were taken using epifluorescent illumination at *λ*ex = 358 nm, *λ*em = 461 nm (for Hoechst 33342/live cell visualization), *λ*ex = 550 nm, *λ*em = 650 nm (for propidium iodide/dead cell visualization) and λex = 495 nm, λem = 515 nm (for Pancytokeratin antibodies/NHK visualization).

### Assessment of colony formation by image analysis

Assessment of NHK colony formation by image analysis was as described previously [7]. Briefly, normal light micrographs of NHK colonies were taken at random using a Leica DM-IRB inverted microscope. A image analysis software (Openlab v.3.0.2) was then employed to evaluate the percentage of the tissue culture area that was occupied by keratinocyte colonies. The fields of vision were examined in 24 wells to obtain a mean±SD (n = 6) for each experiment.
